# Report of Discussions and Proceedings of the Mississippi Valley Association of Dental Surgeons

**Published:** 1852-01

**Authors:** Allen Leslie


### ARTICLE IV.

### *Report of Discussions and Proceedings of the Mississippi Valley Association of Dental Surgeons.* By ALLEN LESLIE.

WE had the pleasure of attending the seventh annual meeting of this Association, held at Louisville, the 11th, 12th and 13th of September last. This, in some respects, was an interesting convocation, and to some, doubtless, may prove a profitable one. We regret the necessity that prevented our presence during the first day's proceedings, and prevented our hearing the address of the president, Dr. Griffith. But we understood it evinced his usual clearness and comprehension.

During the first day's setting, an invitation was extended to the profession in Louisville, to participate in the discussions of the subjects which might come before the Society. Drs. Davis, Dudley and Kirkpatrick being present, embraced the opportunity thus afforded. During the same day's session, Dr. Goddard, in consideration of the high professional standing and moral worth of Dr. Somerby, moved that he be declared an honorary member of the Society. This the Society unanimously agreed to.

In the interim between the morning and afternoon session, the Society, by invitation, dined with the president. The evening session was spent at the office of Dr. Goddard, in the discussion of the queries presented to the Society, by Dr. Drake, five years ago, and from time to time referred to committees. No report ever having been obtained. Of this discussion I can say nothing, not being present, further than that I understood Dr. James Taylor took notes of it that he might make a report in the Register.

*Thursday Morning*, 9 *o'clock.*

Society met again at Temperance Hall.

During this session, were present, Drs. Griffith, Goddard, Baxter, Ulrey, Hunt, Allen, James Taylor, McCallom, Leslie, Dudley, Davis and Kirkpatrick.

Dr. Ulrey being called upon, read a short paper on the subject of atmospheric pressure plates.

On this, Dr. James Taylor remarked. That he had been depending more upon this principle, for sustaining plates within the last year, than formerly, and believed his practice during that time, in this particular, had been more successful. He did not confine himself to any particular form of chamber, but varied it according to circumstances. He had made chambers by enlarging the ruga. Had formed the Flagg chamber, and, indeed, had tried all proposed forms. He, however, preferred the chamber in the center of the arch. He usually made the one he prefers, by placing some wax on that portion of the plaster model, and trimming it to about the thickness of a dime, leaving its edge acute. In this way he was able to strike up a chamber at the same time he struck up his plate, and no soldering was required about it. He has during the past year been using plaster, as the substance to take his impressions, and thought his better success during that time was owing to that fact. And he would recommend it to the Society as a thing altogether superior to wax, for impressions. He knew it was more difficult to use at first, but if gentlemen would stick to its use in full upper sets, in a short time they would not abandon it. He had made out to take some under impressions with it, but here, and in partial sets, he generally uses wax. In using plaster he places a rim of wax across the back of the holder. In the center of the impression before its removal, and after it has set, he inserts a probe, which allows the air to pass into the arch. Then immediately the impression may be removed. He did not think salt necessary in using plaster to make it set quick.

Dr. Allen. Preferred wax as a substance to take impressions. He had tried all the various chambers. He had found those on the ridge very serviceable. He had constructed some under the front teeth, that worked well. He thought an advantage arising from the use of chambers on the ridge, to be the overcoming of some of the evil results of soft spongy gums. He preferred to form the plate by striking up the chamber and plate in one piece as followed by Dr. Taylor. He usually made the plate as large as possible, believing that the larger it was the better it would hold. But he held that much depended upon the articulations of the teeth, whether or not the work would be serviceable. Many failed by setting the teeth too far outside the ridge. He had frequently to correct the work of his young men in this particular, and he thought it a point too much overlooked generally. The Dr. seemed to consider a chamber an essential part of atmospheric plates.

Dr. Goddard's views being called for, he said, he did not know any thing about it. He thought it a mistake to make plates as wide as we had been in the habit of making them the last few years. He had found that wide plates, after being worn for some time, would be found bearing hard upon the center of the arch, creating thereby a rocking motion of the plate, the result as he conceived from the wasting of the ridge. He is now using the old fashioned moderate sized plate, which does not cover all the hard palate. He also prefers wax as a substance for the impression.

Dr. Taylor. Had also found the rocking motion from undue pressure on the arch. He had tried to obviate the difficulty by adding some wax at that part of the plaster model before making the plate, but it did not overcome the difficulty. He, therefore, inclined to the opinion, that plates were frequently made too wide, and that we had better make them in some cases, as Dr. Goddard suggested.

He agreed with Dr. Allen, in the principle stated, that pressure is generally according to the surface, but he thought the plate should not extend further behind the chamber, than sufficient to give a bearing to the edge of the chamber and prevent its being forced into the gum.

In answer to the request of Dr. Leslie, that he would explain on scientific principles, the mode upon which the partial vacuum in the chamber is obtained. The Dr. said, he did not know that he could give a full explanation of the thing. About all we know is, that some how or other, it was done by the wearer applying the tongue and sucking out a portion of the air. In answer to another query, the Dr. said, he used the plaster about the thickness of cream. Dr. Leslie further asked, how, in that case, he got the impression of a mouth with a high arch. Dr. Taylor said, there was always a portion of plaster in the bottom of the cup a little thicker. This he removed, and with a spatula placed it in the elevated portion of the arch; he then inserted his holder containing the balance.

As an instance in proof that a narrow plate would some times be preferable, he stated that he had lately been called upon by a lady having a temporary upper set which was not a good fit. She wished a set inserted on the pressure principle. He took an impression, and the first trial obtained a plate requiring some ten or twelve pounds weight to dislodge it by a direct pull. He attached the teeth and inserted it to his own satisfaction, but practically they did not suit the lady. If she attempted to bite with the front teeth, the back part of the plate would drop. Still she could use the old set in this way without that effect, although a little direct force would dislodge them. He made a second and a third set, but without making them preferable to the old one. He then concluded to make a plate just like the old one, which was narrow. He did so, and they proved more satisfactory.

Dr. Leslie remarked, that to him this was an interesting subject, for he had never been able to agree with the majority in the views they held. This is a matter which has occupied the attention of the profession through our periodicals and otherwise, quite largely and for a length of time, and he had watched the devolopment closely. Conscious that his views differed, he has endeavored to sift the views of others on this subject, in order that the searching might if possible clear up any mistakes he himself may have made. This it was also that pointed the queries addressed to Dr. Taylor.

I have, Mr. President, always been a questioner of the advantages of cavity plates. Hence, whenever I have met with gentlemen who claimed experience in the use of them, I have put such questions as I did to him. I claim not to have had much practical experience in the use of the cavity, simply because I have never been able to convince myself of their advantage, or to have been so fortunate as to be so enlightened by others; hence my course. And if I am pointed in my remarks, it is simply that I may convince or be convinced of error.

My own view of the matter is, that cavities in atmospheric plates will ultimately be abandoned. I found this opinion on the fact, that with but one exception the arrangements of this nature are unscientific in their construction, and that a vacuum cannot be formed in any of them, unless they possess the valve of Dr. Dwinelle. They then become the exception which may be considered scientific in its construction. I have asked Dr. Taylor, as I have others, to explain how even a partial vacuum can be formed in the cavities he and they make use of. And I have found that when the difficulty of explanation was not immediately perceived it was at least felt, and the most I have obtained was, that they judged by the *effects* the cavity produced in some cases upon the gum, that a portion of the air was exhausted; secondly, that such plates adhere more firmly than those without the cavity. And they consequently conclude the air was got out by "suction." Now, sir, I think that gentlemen allow themselves to be led astray by *appearances.* I conceive it to be no proof that there has been a partial vacuum in a cavity, because you find on removal of such a plate that the gum over such cavity is swollen. This *may* be the result in a mouth where the gum is in any way spongy if having a cavity there.

That portion presented to the cavity is not subjected to the pressure the portion is which the surrounding plate lies in close contact with, it is, therefore, left free to develop fully its spongy tendency by protruding into the cavity. And this tendency, be it noticed, is increaced by the *surrounding* pressure. So that you see, sir, I must take part of their evidence, and claim it as another proof that the cavity plate must be abandoned.

I hold further, that cavity plates must be abandoned, even were I wrong in assigning the swelling of the gum into such cavities to the absence of pressure there; because, I hold that if there is a vacuum, be its degree what it may, the tendency must inevitably always be for the gum to fill up the cavity. And I feel assured, by analogy, that where the means exist of forming a vacuum in such cavities, the gum would ultimately fill them up, unless such means of preventing it are adopted in Cleaveland's chamber, which may be viewed as a chamber beyond the ordinary chamber, having very small openings into the second, which arrangement, the patentee himself says, is to prevent the gum swelling so as to fill up the second chamber. This clearly proves one of my positions, that *if* a vacuum is obtained in ordinary cavities it will in time be filled up by the gum.

If we examine the actions of an air pump exhausting the air from a vessel on the lecturer's table, we perceive he attaches the pump to a truly turned metallic plate, in the center of which we observe a small opening which communicates with the pump chamber. We understand at once the object of it; it is for the egress of the air. If the edge of the glass vessel is not perfectly true so as to form a *perfect* joint with the plate, he uses a rim of buckskin to effect this, so essential is it that the edges of *his* cavity should fit close *all round* when he *commences* exhausting their chamber. Not so, the dentist who uses the cavity, he tilts the vessel (cavity) to one side and

"sucks" the air out. We have another illustration of the partial or complete exhausting of a chamber in the act of cupping. These cups, in which the air pump is used to exhaust the air. These have a valve on the top, and we see at once how the air may be exhausted, it is done precisely on the principle of Dr. Dwinelle's chamber. We have also the old fashioned cup, in which a flame is inserted for the expulsion of a portion of the air. Here again we see the mode; not so in the cavity practice, and until we are shown a better explanation than has yet been given, we must hold that they have not any more pressure by means of the cavity than can be got without, and I question if they have as much as exists in a well formed plain plate.

A question, which I think of some importance to determine, as nearly as we can, is, what *amount* of atmospheric pressure is requisite on an upper set of teeth, in order that they be useful. Some men there are who promise their patients from five to twenty pounds, as it may be desired, or as they think the case demands. To hear them talk, one would think it was as easy to do this as to make the chamber. But for my part, I never say any thing of pounds to my patients, all I promise is a useful set of teeth; this is all that is needed. It may not be startling enough for a striking advertisment, but as I never aim at such things, it matters not to me. I trust always to redeem my pledge, by using the plain plate; and here let me say, that it must, I think, be within the power of all dentists of experience to refer to sets of teeth they have inserted with the plate, void of any cavity, which gave entire satisfaction to the wearer, without the aid of springs. And I believe it may be said of such cases, that they are preferred by the wearer as well as the operator to those with cavities.

The amount of pressure on a well filled and properly articulated set of upper teeth, need, I think, but little exceed the actual weight of the operation, and the pressure exerted by the tongue in articulation. Where the plate is properly adapted, and there is any thing of an arch in the mouth, and the plate can be brought over the ridge, mastication but increases that pressure on the exposed surface of the plate. Of such a plate, of course it is a prerequisite that it should stand pressure in any part, without dropping at the opposite point.

We all know that some plates we make take hold, so to speak, of the gum better than others. This irregularity is attributed by some to various causes, peculiar form of the mouth, sponginess of the gum, &c. &c. When I meet with a plate that does not stay up, I lay it to myself, not to the mouth, to a failure in my impression, and set about taking another. I do just the same when it rocks on the center of the arch, I view it as my fault. I may have failed in gaining the impression from pressure against the lips in removal. I think more harm results from these soft parts, than from those on the ridge, which some gentlemen fear so much. Having obtained a good impression, I consider there is no difficulty in making a plate which will be retained by atmospheric pressure.

This atmospheric pressure I conceive to be obtained by an approach to a perfect *adaptation of the plate to the mouth.* The more perfect this is, the greater the pressure. Such a plate, when placed in the mouth and forced against the gum, displaces the air that is in contact with that surface. And *thus* the balance of pressure is created upon the exposed surface of the plate, and this it is, which retains it in place. Hence, simple occlusion of the jaw is *sufficient* to restore the pressure when the plate inclines to drop.

This, I conceive to be the true theory of atmospheric pressure, as applied in dentistry. Consequently, I cover as much surface as possible, when I want this pressure. This is in accordance with the well known principle alluded to by Dr. Allen, that the pressure is according to the extent of surface.

It is in accordance with this theory, which I have endeavored to develop, that I conceive the artificial enlargement of the ruga, and all forms of chambers not possessing the valve of Dwinelle, as worse than useless, from said cavities being reservoirs of air, by which the plate is sooner separated from the surface of the gum. And as a matter of course, if the air is not partially exhausted from the chamber, there is not as much pressure on the plate as if it was not there.

I object, then, to the use of chambers. First, because they are not what they are supposed to be, partial vacuums. Second, that if they were, they would to that extent produce swelling of the gum into the cavity. Thirdly, because they are unnecessary, the simple plate being sufficient. I throw these thoughts out, observing, that if there be error in them, it will be shown by those who perceive it.

Next, sir, a word or two on the impression. It is claimed by those who prefer prepared gypsum as a medium to receive an impression, that its chief advantage consists in the perfection with which they obtain an impression of the soft ruga and spongy ridges, without any displacement of the soft parts, there being no pressure required in its use. While in the use of wax, these parts are more or less displaced.

Of plaster, I would say, that if it were possible to use it under circumstances requisite to its most perfect application, that *it* would undoubtedly be preferable. But I have yet to learn how it may be so managed. To obtain with it a *perfect* impression, it should be used as thin as the medalion maker employs it. This is much thinner than Dr. Taylor employs it. You will now, sir, perceive the force of my inquiry of the doctor as to how he obtained an impression of the elevated arch.

Here he finds it necessary to use, shall I say, a piece, it would be near, if not quite the truth. At least, it must be considerably thicker than cream. I had thought, sir, and still do not see otherwise, that plaster should be used just as it begins to set. Because, if used in the liquid state, it must follow the law of liquids, viz. seek a level. Now, sir, I question whether in either of these states, a more perfect impression can be obtained with it than with wax. Again, sir, is it a fact that it can and is used without any pressure. If so, a steady hand must sustain the holder, an equally steady one the patient's head, as the *least* motion during the setting of the plaster, *must* change the impression. I will, sir, frankly acknowledge, that with wax it may be impossible to get an *absolutely perfect* impression. But, sir, I think that with proper care, your success will be quite as great with wax as with plaster as *now*

used, and while the advantage is clearly in favor of the plaster, the simplicity of wax must give it the preference. I make use of pure yellow wax, softened in water, not above 140° F. It should never be allowed to melt in the surface. It is also best to use it but once, it is then tougher, not to mention cleanliness.

Another thought which has been presented, of which I could not see the philosophy. It has been asserted here, and I have heard of it elsewhere, that if you insert a probe through the plaster or wax, until it touch the elevated portion of the arch, you could immediately remove an impression, which, without such a course, would have been destroyed by the force necessary to remove it at the same period. I say, sir, I cannot understand the working of the probe. It may be ignorance, but it appears to me, that if I was to fill a vessel with wax or plaster, and turn it bottom up, and insert a probe such as the gentlemen uses, it would only allow air to pass to the surface the probe touched.

By what means the air is to pass between the vessel and the wax, or between the gum and wax or plaster, radiating from the small opening, I cannot so clearly see, especially as its effects are said to be immediate. If it were claimed to be a slow process, I would admit it may be of some utility. Or if the plaster or wax had not been placed in close contact with the highest portion of the arch, I could see how the external opening would operate upon the confined air in that cavity, but in the way claimed I cannot. I could see how, if it were such a fluid as water that filled the supposed cup or arch of the mouth, that the insertion of a tube which would pass air to the upper surface would radiate *immediately* over said surface, because in this case, the fluid would immediately descend, not so in the other. In my own practice, consequently, I make no use of the probe. Not because my impressions do not adhere as firmly as those of others, but because I conceive the mode I adopt, of overcoming the difficulty, as superior. My mode of procedure is based upon what I believe to be a fact, viz. that the soft parts (buccinator muscle and mucous membrane) act the part in the impression that the rim of buckskin does under the air vessel. It makes the adaptation more perfect.

My first step in accordance with this view, after the wax has set is to raise these soft parts, first on one side and then the other, repeatedly if necessary, allowing the air in this way to gain access to the whole rim of the impression. The result I find to be that an impression which before would have been destroyed in the attempt to remove it, now may be removed safely. I can feel it give way. In the language of others, "it will presently drop."

Dr. Griffith said, he had been in the habit of using the probe and found it very serviceable.

Dr. Taylor admitted, that the soft parts named by Dr. Leslie, played an important part in the adhesion of the impression, and he always raised these also, so Dr. L. had described. Still he contended, that the probe was indispensable, and that the air did pass from the opening it made, over the surface of the arch.

He was certain his views respecting the great advantage plaster had over wax, would be found to be sustained, if gentlemen would try it more. One thing he would say for it, and that much he could not say for wax. He would undertake to get a plate that would stick to the mouth, from the first impression. If he could not do this with any mouth, he would forfeit fifty dollars. He, however, believed the day was approaching when cavities would be abandoned, and the simple plate be proved sufficient for our purpose, for he must say that he had inserted many sets without the chamber, and that on the whole, he and his patients preferred such, when he could get them, to those with the chamber.

The address of Dr. Baxter, on the use of palladium, being next in order, at his request, further time was granted him, and he was appointed one of the essayists for next year.

Drs. Davis and Kirkpatrick were, on presentation, by the examing committee, elected members of the society.

Dr. John Allen, who was appointed at the fifth annual meeting to prepare an address on the subject of artificial gums, was next called upon. The Dr. arose, and stated that he had not prepared an address on the subject, and if he presented any thing it would have to be extempore. The Dr. informed the society that he had been experimenting a number of years, endeavoing to perfect this thing. He had been at great expense before he had arrived at the state of perfection he had now reached, and he felt convinced he had now got all that need be desired in this way. He had discovered a method by means of which single teeth could be surrounded by a silicious substance, which would stick to them and to the plate firmly.

The Dr. held that his improvement would entirely change the whole practice of mechanical dentistry. The advantages he conceived to be, great expedition, great strength, perfect cleanliness ; as it is impossible, he says, for even moisture to get between the plate and this silicious cement. It is also susceptible of greater beauty of finish. It is also of especial superiority, when atmospheric pressure is desired, as the plate undergoes no change whatever in the fire. He has also tested the matter practically in the mouth, and was fully satisfied of its usefulness. These were the main points claimed by Dr. Allen, for what he claimed as his invention.

Dr. Allen presented several pretty specimens, and eulogized his improvement very effectually. This was the amount of his address, and all we are constrained to say, which appeared to be his object, for when he had taken his seat we knew nothing more about the subject than when he arose. We had heard no new principle elucidated, no new fact explained, none of his materials even *named.* He also informed us that one of the most essential parts of the discovery consisted in a means of retaining the teeth in place, during the fusion of the cement. This he claimed to have discovered, but did not make known.

We should not say less than that we considered it trifling with the time of the Society, for the Dr. to come before it thus. But so thought not all of the Association, as immediately the following preamble and resolution was offered by Dr. Goddard :

*Whereas*, Dr. J. Allen of Cincinnati, has been for several years engaged in prosecuting a series of experiments, of which we have been cognizant, for the purpose of acquiring principles by means of which an artificial gum could be formed upon mineral teeth, and metallic plates, in such a manner as to unite them firmly to each other, and thereby render more perfect the present method of setting artificial teeth on plate. And whereas, the results of his experiments have been highly satisfactory to the members of this Association, as exemplified in the specimens he has exhibited. And believing it due to any member of our society, who devotes his time, money and talents to the advancment of any particular branch of the profession, so that benefit may result therefrom, that some action or commendation is necessary.

Therefore, in view of the great benefit which must result to the profession, and the public generally, from the indefatigable exertions of our brother, Dr. J. Allen, in producing a mineral substance, by the use of which artificial teeth may be more perfectly placed in the mouth, and made to resemble the natural organs of mastication.

*Resolved*, That this society award to him a gold medal.

This being seconded by Dr. James Taylor, the president undertook immediately to take the vote. But one of the members desiring to discuss the matter, it was, on his motion, laid on the table until the afternoon session.

The society then adjourned.

*Afternoon Session.*

The President announced the first business to be the consideration of the motion laid on the table before adjournment.

Dr. Taylor desired now to withdraw his second, and offer another resolution as a substitute.

After some discussion on the points of order involved, further action was, on the suggestion of Dr. Leslie, waived, until the mover, Dr. Goddard, should be present.

A short time was now allowed in hearing some remarks of Dr. Davis, on the use of palladium, as a base for teeth. The doctor claimed for it a preference over gold, as regards oxydation. Also greater strength, and consequently less weight in a given piece of work, than if made of gold, and a saving in cost. He claimed for Dr. Childs and himself the credit of having introduced it into practice. This they done in Philadelphia.

But as the doctor intends preparing a paper for publication on the subject, fully explaining his practice and experience in the matter, we defer further remarks.

The consideration of the preamble and resolution of Dr. Goddard, was now resumed, he being present, when, on motion of Dr. Taylor, they were referred to a committee of three, to report in the evening. The chair appointed Drs. Taylor, Goddard and Leslie, said committee.

Dr. Goddard presented the name of Dr. Somerby, as a candidate for active membership in this society. A ballot having been had, he was declared unanimously elected.

Adjourned to meet at the office of Dr. Somerby, at 7, P. M.

*Evening Session.*

Committee of three called upon to report.

Dr. Goddard asked for the committee further time. It was allowed until the morning to prepare its report.

The evening session was mainly spent in discussing the treatment of teeth in plugging, after the exposure of the nerve.

Dr. Allen related what he could call to mind, of the discussions just held in the American Society on this point, and stated the aphorism adopted on this subject. He preferred to cut out the nerve at once. He uses a small piece of hickory wood; but his own experience in the practice, was not as encouraging as that of many members of that society.

Dr. Griffith called the attention of the society to his mode of treatment, which he had spoken of a year ago. When he exposed the lining membrane of a tooth, he wiped the cavity out carefully, and placed next the membrane a small portion of cotton, partially saturated with kreosote; over this he plugged, and he experienced no difficulty afterwards. He had removed many fillings of this kind, and had found that the kreosote was absorbed, and he considered that it had excited a degree of inflammation which resulted in the formation of a new layer of bone, as a protection to the pulp and membrane.

Dr. Goddard had no experience in plugging over nerves, he preferred excision. Had plugged many teeth in the latter mode, and had found it to work well, and believed the remaining portion of the nerve took on a healthy action, and in many instances deposited a protecting layer of bone.

Dr. Somerby had always viewed this from a physiological point of view. He had early been grounded in correct pathological and physiological principles and anatomical facts, and he objected to this plugging of dead teeth, because it could not be done unless some of the laws of physiology were outraged. He could not see how arsenious acid could be so used as to destroy the nerve just to the point of exit from the fang, and there stop. He could not so nicely arrange it, as some gentlemen appeared they could.

He thought if its influence reached the point, it would be felt beyond it, and produce disease, ultimating in the extraction of the tooth. The doctor believed that more injury had been done by the announcement that teeth could be saved after the exposure of the nerve, than had resulted from the use of amalgam. His experience in the extraction of teeth, which had been treated in this way, was quite extensive ; he, perhaps, saw more cases of failures, than those did who operated thus, and he thought we ought to be careful that in adopting this practice, we should base it upon extensive experience. In illustration of what *may* result in constitutional derangements, from the presence of a small amount of decomposing or poisonous matter, at the point of a fang of a tooth, the doctor referred to the poison of the rattlesnake, the virus of the cow pock, the wound in dissecting, and the sting of a bee. He had also found in this kind of operation, the most inferior plugger was more apt to avoid immediate trouble, than the most finished workman, because his mushy fillings allowed gaseous, or more solid fluids to escape through them, while the solid fillings make them seek egress through the fang.

The society, before adjournment, went into an election for officers. Dr. Somerby was elected President, and James Taylor, Editor of the Register.

By report of the editor, it was found the cost of the publication was some fifty dollars in excess of his receipts; an order on the treasurer was drawn for the amount. Dr. Taylor informed members of the society, that he would, during the coming year, conduct it at his own expense, and with this understanding, it was continued under his editorship.

Adjourned.

*Friday*, 8, A. M.

Committee on preamble and resolution being called upon for a report, presented the following majority report:

"The committee to whom was referred the preamble and resolution of Dr. Goddard, in relation to Dr. Allen's improvement in the method of attaching teeth to plates, submit the following report:

"That they have examined the teeth cemented to the plate, by Dr. Allen, and have subjected them to the following tests: they have tried their strength of adhesion, and believe that no ordinary force, such as is used in masticating food, &c., will loosen them from the plate; indeed, so far as they can judge, the adhesion to the plate is much the same as that effected by the use of solder, hence, the entire base of the teeth being secured by the cement, there is greater solidity, and no room for the lodgment of particles of food about the teeth, thus forming a substitute for block work, possessing all the advantages of block work, with more strength and security to the plate.

"They have subjected the gum to the action of nitric and sulphuric acids, and after the pieces had lain over night in these acids, they found no appreciable effect made upon them, although the acids were in a concentrated form. The committee are satisfied that this mode of securing the teeth to the plate, recommended by Dr. Allen, possesses cleanliness and strength, and so far as we can judge, durability.

"The committee would remark, that in using this cement, the plate used by Dr. Allen is platinum and pure gold, alloyed with 4-100 of platinum, and this greater purity of metal, which more effectually resists the action of the secretions of the mouth, they regard as advantageous, because it secures the public against the use of impure gold in mechanical dentistry. In view of the labor and expense to which we are satisfied

Dr. Allen has been subjected, in bringing this improvement to its present state of perfection, and the advantage to the profession which its adoption we think will insure, we therefore recommend the following resolution :

*Be it Resolved*, That Dr. Allen deserves all commendation for his indefatigable exertions in thus developing, and making available, a new and important improvement in mechanical dentistry, and that we recommend this improvement to the profession, as worthy of their attention.

JAMES TAYLOR,
W. H. GODDARD,
*Majority of Committee.*

On motion, this report was accepted. When Dr. Leslie presented the following minority report:

Report of a minority of the committee to whom was referred the preamble and resolution of Dr. Goddard.

Your committee would report, that it has ascertained from Dr. Allen, that it is his intention to procure, if possible, a patent for said mode of work, and that he has filed his caveat in the patent office for the same. This your committee would deem sufficient reason to advise the society not to grant any award of merit in the case, inasmuch as he looks for a reward in the sale of patent rights. Your committee still further feel constrained to object to the award, because the principles which led to the formation of this and kindred societies are inevitably subverted, when the right is recognized in any of its members to patent their improvements in practice.

It is also to be objected to, because it is contrary to the expressed will of the society, as shown by several resolutions to be found in its published minutes, as well as the public teachings of some of its prominent members. It is also not only not sustained by the most enlightened dentists every where, but is by them most decidedly disapproved.

Farther, your committee have had no proof of the practical usefulness of this mode, no cases having been brought before it which had been subjected for any length of time to the friction of mastication, and your committee would suggest that one year, at least, is requisite to test the mode in this respect; and that until that time expires, no resolution approving it should be passed by this society.

Again, your committee have had no insight into the matter afforded them by Dr. Allen. He has come before it, as he has before the society, with the matter as a secret. No queries are answered, no light is given, which was not already in the possession of all or a part of the committee.

In view of the foregoing facts, your committee recommend that you do not accede to the proposed award of a gold medal. And further, your committee recommend the adoption of the following resolution:

*Resolved*, That it is now, and always has been, the sentiment of this society, that it is derogatory to the professional character of any of its members to patent any instrument or improved mode of practice. And, inasmuch, as the forbearance of this society has heretofore been misunderstood, we do now declare, that for any member of this society to patent any instrument or improved mode of practice shall be deemed sufficient cause for expulsion.

Respectfully submitted,

A. M. LESLIE,
*Minority of Committee.*

Dr. Leslie moved the acceptance of this report, and Dr. Goddard responded with a second.

Dr. Leslie opened discussion upon it, by saying, that he would substantiate that report by a reference to documents no one could gainsay. He did not wish the society to suppose he had spent much time in the preparation of the report, as the probable necessity for it had only become apparent a short time previous to this morning's meeting. Further, he would say that he would present but a small portion of such documentary support as might be produced in proof of the correctness of the report, had more time been afforded for the same.

First, this report advised that you refuse the gold medal, because the claimant of the improvement intends patenting the same if he can, this he acknowledges. I will now endeavor to show you, from your own acts, that this society can give no countenance to the proposition awarding a gold medal for any patent improvement. That this is no new proposition here, I call your attention to the published minutes of the first annual session of this society. On page 12, we find the following, presented by Dr. Challen:

"*Resolved*, That the discoveries and improvements known to the members of the dental profession, are, and ought to be, considered as the common property of the profession."

Here, then, we see that this was one of the first subjects which the association thought necessary to come before it, and it thus early threw its influence against patents. But I propose still further to show, that even this position was deemed not decided enough for this association to hold in this matter. For they had found that even in the face of this expression, one of its officers, viz. Dr. John Allen, of Cincinnati, here presented what he conceived to be a very important improvement. And I will venture to say, that the respected mover of the resolution I will now read, designed to apply it, if adopted, to the very case I have mentioned. I will read now from the minutes of the second annual meeting, page 7:

"The following resolution was offered by Dr. Edward Taylor:

"*Resolved*, That any member of this society, who may patent any instrument or improved mode of practice, may be subject to expulsion from the society."

In discussing this it was "especially contended by Dr. James Taylor, that while a piece of mechanism applied to the arts, and which can be made and sold, wholesale and retail, so that all who wish may buy and use, and the inventor receive his reward without injury to the public—might, with propriety, be patented; yet, a principle of that science which claims for its object the amelioration of the ills of life, which seeks, on all occasions, to be known for its usefulness, which we as a society have banded together, as brothers, to elevate, should never be thus trammeled. No letters patent should be thrown around her outstretched pinions. Naught but the diction of the great Almighty should be interposed to say, "thus far shalt thou go and no further."

On motion, the resolution was then laid on the table until to-morrow.

It will be seen by the sentiment of this resolution, and the remark appended, that there was a decided liberal spirit manifested and upheld by the society, the majority of the speakers being in favor of the resolution. There can be no doubt, that if the motion had been pressed to a vote, the resolution would have been adopted. But without wishing to cut a member off without a chance for reformation, it was thought best for the present, to modify their action.

Consequently, Dr. E. Taylor the next day presented the following, which you will find on page 11, minutes of second annual meeting :

"*Resolved*, That viewing ours as an important branch of one of the liberal professions, we feel bound to disapprove of any member of this society patenting any instrument or mode of practice pertaining to our profession, and that further action on this subject be deferred until our next annual meeting." Adopted.

Such, gentlemen, is the position this society now stands in before the profession. It has declared, that the member who shall patent any instrument or improved mode of practice does that, which, we *feel bound* to say, merits our condemnation.

And now I ask, are you ready to abandon this position ? I hope not, but I must say, I fear for the result. It seems to me this is just the time for men to stand by it. This is just such a case as it should be applied to. For what are the facts ? A member, who has been an officer of this society from its commencement, who has known its laws, and who has sinned against the principles of this association once already, but was forborne with, in hopes that time for repentance would work a change, comes again before the society with what he claims as his improvement, and which he says, will completely revolutionize the practice of mechanical dentistry, and tells us what, pray, of it. I say, literally nothing. True, he says, it is a great improvement. That the teeth are set in a silicious substance. That it fastens them firmly to the plate without soldering. That *he* is satisfied they will stand the friction of mastication. That no moisture can get between the plate and the cement, and that there is no possibility of shrinkage or warpage in the plate in this method of work. But so far from attempting a development of *how* he possesses all these desideratums, not a ray of light is given, unless his answer to a member might be considered such. He was asked how he so certainly overcame warpage. He answered "it was by the use of plaster and a—a—another substance."

Now, sir, I hold that there is as much light contained in his advertisement as there is in his addresses on the subject.

Such, Mr. President, is the result to the society of waiting two years for his paper on artificial gums. He has come before your committee as he has before the society, presenting this whole matter as a secret, and I am astonished that gentlemen who have, in former years, so explicitly and eloquently expressed themselves, should now be willing to vote a gold medal as a reward for this improvement. I call upon them for their reasons for this change of action. Certain am I, that if this society has settled any one point of action, that point is, that it disapproves of patenting such an improvement as is now under discussion.

Again, it is fatal to all the principles of our association, to encourage the course Dr. Allen has taken, and still intends to pursue. I had thought, sir, that our object in forming this society, was to elevate ourselves and the profession we follow, by individual improvement. And that we should meet annually for the *free* interchange of ideas between the members. That these ideas and improvements, if deemed of sufficient importance, should be sent, broadcast, through the profession, and that in this way we would labor to elevate and improve the knowledge and practice of the profession at large. And not that we should come together yearly to eulogise our own modes of practice—hint at the desideratums we have obtained, and the points in which our (secret) mode of practice excels all others, and vote gold medals, as rewards for *such* valuable information. Sir, give countenance to the proposition which was placed in the hands of the committee, or adopt the majority report, and you establish a precedent, which will, if followed up, eventually result in the destruction of this society. Sir, it undermines the very corner stone of our whole fabric. It says most emphatically, you *may* patent any of your improvements in dentistry, even should this improvement entirely change, and supplant the ordinary mode of practice in an extensive branch. We say, Mr. Patentee, we will give you that which will be of essential service to you, in your legal effort to clip the "pinions" of the profession. In order to gratify you, we will repudiate all we have formerly done. Yield our position before the profession, and retract our words, our motive being only the encouragement of your laudable pursuit.

Mr. President, how shall we be viewed by the profession, if we thus stultify ourselves.

First, then, I repeat, we object to the preamble and resolution which would award a gold medal, on the ground that it is contrary to the expressed will of the society, and subversive of the principles of its foundation to give *any* encouragement to patent improvements. Of this, I trust you are satisfied from the evidence I have adduced. But further, sir, the report I have presented, claims that it is contrary to the views entertained by the prominent members of this and kindred societies, and of the profession at large, who claim for it that it is a branch of a liberal profession, any countenance to those who shall patent improvement in dentistry.

This, we also find abundantly evident in addresses and other papers, as set forth in the periodicals of the profession. Time would fail me to quote a tithe of the matter which might be collected on this head. I will confine myself to some quotations, going to show, still further, the views of some gentlemen, now members of this society. I do this to show that I do not misunderstand their position, and, consequently, do not misrepresent them. First, let me read you a few lines from an address of Dr. E. Taylor, delivered at the third annual meeting of this society, vol. 1, page 6, Dental Register. "We are banded together for the purpose of elevating the standard of our profession. We here form a common fund, into which each member casts, of moral and professional character, his all. The aggregate, therefore, forms our professional force, and that force will be, of course, greater or less, as may be our individual contributions. That each of us have a large amount of capital thus invested, should be our highest ambition; for thus invested, it yields largely to the benefit of the community, and insures an ample return of interest to each individual stockholder."

We violate the intention of the speaker, but little, I think, when we connect this sentiment with the resolutions already quoted.

I shall read next, sir, the sentiment of Dr. B. B. Brown, late of St. Louis. At the time of writing, one of the editors of the Dental Register. Turn with me to page 45 of vol. 2d, of that valuable work, and we will see what he says on the point.

"The enlightened dental profession must view with suspicion, if not with extreme disgust, every effort on the part of those practitioners who, by means of secret remedies, or "*patent penny whistles*," endeavor to construct fortunes by such mercenary practices as inflict shameful wrongs upon a noble profession, which they, unfortunately, have disgraced in the membership of being so base. They have, undoubtedly mistaken their calling; they should forthwith devote their valuable time and eminent talents to the more appropriate and congenial pursuits of peddling patent washing machines, wooden nutmegs, or white oak cucumber seed." St. Louis Ed.

Bear with me, sir, while I still further read from our Register. I ask your attention to an extract from a valedictory address by Professor Mendenhall, of the Ohio College of Dental Surgery—an honorary member of this society. He is giving what he conceives to be, wholesome advice to a class of graduates, young men, who are supposed to be inexperienced, and in need of being instructed in the principles which govern associative action. On page 113, vol. 3d, he says:

"In regard to the relations you hold towards those honorably practicing your specialty, the same general conditions must govern. While a fair and honorable competition is allowable, nothing can be tolerated which savors of the spirit of quackery. Every man, before he can expect your fraternal confidence, must practice his profession in an open, candid manner. He must make no exclusive, high-sounding, and noisy pretensions; possess no knowledge but what he is willing to communicate freely to others of the profession of medicine; and deal in no specifics or secret remedies, or plans of practice. This course you have a right to expect of others, and they have a right to demand the same of you. A contrary one must result in a want of confidence, and is mean, undignified, unmanly, dishonorable and disgraceful in the highest degree. The indignation of every member of the profession should be visited on any man who arrogates to himself knowledge and modes of treating disease that he is not willing to scatter abroad among his professional brethren. A sordid spirit of that kind should be rebuked, no matter what qualifications its possessor may have. Every man, also, upon entering a profession, places himself under obligation to that profession to contribute to its common stock of knowledge, and to aid in its elevation and advancement, by adding something to it not before known. Our whole science being progressive, we must assist in promoting its onward march—each in his proper department must exhibit a commendable spirit of industry. We must neither be too indolent, too cowardly, or too proud to aid in fulfilling the mission which destiny has marked out for that profession which has science for its foundation, and the good of mankind for its great object."

Sir, words need not be multiplied to show the application of of this extract to the present case. If its edge is not felt, such is the obtuseness of feeling, that it would be folly to sharpen them, if it were possible.

But what says Professor Taylor, in his valedictory to a smaller class. Listen, sir, while I read from Dental Register, vol. 2, page 122.

"If the prowling, hydra-headed monster of charlatanism rears up his many heads, to mar her beauty, and sully her good name; with the sword of truth, guided by an arm of Hercules, sever them off, and continue to strike until none reappear, and dentistry, as a science, stands forth pre-eminently beautiful—perfect in every part—the admiration of all.

"Let the ignorant, let the depraved, and the vile, act the part of the quack—but, oh! never let him who hath drank at the fount of knowledge, who hath ever bathed his temples in the refreshing streams of science, or inhaled the fragrant odor of a clear conscience, stoop to her foul embraces.

"Her touch is pollution—her course leads downward and downward—along her path are strewn the victims of her avarice; the shrieks, groans and agonizing cry of the dying, continually pour forth her requiem, and tell but too surely her work is destruction. May she never find a resting place in our profession."

There, sir, is eloquence. Eloquence which must have been inspired by circumstances differing from the present. His "outstretched pinions" could have felt at that time, none of those trammels from letters patent, which is now thrown around him, and which I fear, he is willing now to abandon others unto. But, Mr. President, I must cease quoting; enough has been produced to show the sentiment, heretofore, of some of the members of this society.

I trust, that from what has been said, the members of the society will see that they cannot consistently with their own action and teaching, vote for the resolution awarding a gold medal to John Allen, or any man, for a *patent* improvement.

Another point the report claims, is, that the sentiment of the prominent members of the profession at large, is decidedly opposed to patenting improvements in dental practice. Have I, sir, over-stated the fact. I think not. For not only do they hold this view, but they hold, also, that it is the duty of each individual member, to labor assiduously to improve his own practice by modifications of old modes, but also by the invention of new ones, and that when such changes are *found valuable*, that he seek to elevate the profession in public esteem, and in fact, by improving the general practice thereof, by communicating freely with his brethren, on all such improvements.

Time would fail me, sir, here to speak of a tithe of what has been done by men worthy of our esteem, thus to simplify and render perfect the various operations of our profession, and I know no one here or elsewhere, who is not ready to adopt their improvements or who is not indebted largely to such.

But, sir, as this discussion has been put off until the last moments of our session, and time admonishes me to be as brief as possible, and as action speaks louder than words, I will, for the present, waive any further quotations, and touch on only a few prominent facts which have suggested themselves to me within a short time. First, then, I would call to mind the professional career of the honored and lamented Nasmyth. His life was truly a life of sacrifice to the elevation of the profession. He was one who had few peers in the cultivation of that branch of science to which he devoted himself. His contributions to our stock of microscopic investigation, will long hold a preeminence. He pursued his profession with an ardor and enthusiasm that none can excel. It was not for self he labored, but for you, sir, and you, yea for all of us, for nothing less than the benefit of mankind as a whole. In the midst of business, lucrative and extensive, he found the time, yea, rather took it, took that which was of value above what you can estimate by dollars; that which was essential to his health; the hours in which he should have rested, and devoted them to scientific research. He proposed to throw no letters patent around the "pinions" of those whose impulses might lead them to try this same flight. To all such, he says, pursue it. Here I give you freely my chart to guide you, my lamp to light you, freely you have received, freely give. Hold, says one, he is an author, and as such receives his reward in the shape of profit on his works, secured to himself by his copyright. To such I reply, that no man in lucrative practice, such as his was, can be paid for the time spent in such labor as he performed, from the sale of his works. And further, there can be no comparison between this case and that, because if a patent, such as we now have in view, is carefully drawn up so as to cover principles, *that* especial feeler is shut up from all attempts at gleaning a cultivation, not so the other, the mind is not only left untrammeled, but united to efforts of genius. We further know that in the case of Dr. Nasmyth, cerebral disease and premature death was the result of his efforts to prepare for the press a work since published. Think you, that if money was his aim he had not a better and easier mode of acquiring it? undoubtedly he had; but Nasmyth lived not for himself; had he, few of us would have heard of him. His name and fame shall live when such patents as this are forgotten.

Authors, such as Fox, Hunter, Fitch, Koecker, Brown, Harris, &c. &c., could be adduced, all going to show the development of the liberal spirit, but to name them is enough to the wise. No one here dare deny that this has been the result of our ablest authors writing on both sides of the Atlantic. Much, however, has been done in our own day, and by men still living, that ought to be here noticed. Let us look at some of those things we are every day using, and which might have been made the subject of patents.

One of the most accomplished dental surgeons of our day is Dr. Hullihen, of Wheeling, Va. Few among us have done more, if as much, to elevate the profession in the eyes of our medical brethren than he has by his practice, whether we view that strictly dental, or his operations on soft parts in close proximity with the teeth. What has he done? He has given us Hullihen's screw forcep, an article which he might have patented, and which would have yielded a handsome return for the labor bestowed in getting it up, but he *would not.* He has also made various improved instruments for the operation on cleft palate, none of which he has thought of patenting. But in the true liberal spirit of our worthy brethren of the medical profession, he publishes all unreservedly. He has also done the same with his experiments for the cure of tic douloureux, which present some very peculiar features of treatment.

An improvement which has been highly lauded by professor Taylor, during this session, for the perfection with which it enables him to insert artificial teeth, is that of plaster as the medium to obtain an impression. To whom is he and the profession indebted for this valuable mode. We must, to the best of my knowledge, give the honor of its introduction as well as the honor of *not* patenting it, to Drs. Dunning or Dwinelle, it may be due to both of them equally. Again, sir, we have the opinion of the profession on patents, shown in the controversy on the Lethean patent. Some would say that Dr. Morton was compelled by the sentiment, of both medical and dental profession, to abandon the unprofessional course he had followed; but it may be both wisdom and charity to admit that the doctor was *convinced* of his error, in part at least, by the arguments adduced by his opponents. Another matter which has been made the subject of several patents is the cavity plate. And who of this society, I will ask, has not in practice refused to acknowledge the rights of the various patentees of chambers. Gentlemen tell us they have tried *all* modes, they must then have tried patent ones. Have they paid for the right? I venture to say they have not, and do not intend to. They do not recognize a moral obligation in the matter; they will fall back, when necessary, on the chamber of Dr. Flagg, or some other liberal inventor, and still we will hear of the necessity of encouraging and rewarding the patent inventor; verily men change as well as times.

We have heard much lately of the value of arsenious acid as an agent to destroy the exposed pulp of a tooth. This also is a thing which might have been patented. But Dr. Spooner was too much a medical man not to see the illiberality which such a course would manifest, therefore, he *gave it* to the profession. But, sir, if I were to dwell on this head, I would weary your patience with the recital of that which every dentist who has read must be familiar with. In a word, I would ask you all, particularly the younger members, where did you get your knowledge? have you each and all discovered the modes you practice? are you not rather indebted for nearly all, if not the whole you know, to others. Have you not often felt thankful for the timely advice of your seniors. Do you not rejoice that the day is gone by, when to announce yourself in the office of a dentist as a dentist, was tantamount to an invitation to him to close his operating case. For one I am, and I am glad I never experienced such treatment, and for one, let others do as they may, I will resist every thing that looks like the return of it, let it come howsoever insidious, or under the auspicious patronage of men however high in professional places. For one, I say, let us march onward, not backward.

I believe, Mr. President, that I speak the truth when I say, that a more thorough knowledge of the science of dentistry can now be acquired in two years, if the best means are taken advantage of, than could in ten, if not twenty, years, by the older members of the profession.

The reason is, that in their young days the ruling principle was *secret improvement* and secret practice. With few exceptions each had to teach himself. While in our day the ruling principle is the *liberal instruction of each*, *no secret improvement*, *no secret practice*.

To the American Society and its founders there can be no question we owe much, very much. It is the parent of all the associative advancement the profession has made. It was the lap from whence the seeds were scattered, the fruits of which both old and young, but especially the latter, are now gathering. By it was issued the first dental periodical, to be used as a vehicle by the profession for the interchange of views. It was one of its prime movers that gave life and being to the first dental college.

To the impulse given by the American Society are we also indebted for the formation of state societies and the publication of various periodicals, devoted entirely to the discussion of dental practice, all of which, I believe, have uniformly sustained the principles involved in the minority report. And for our journals and societies to act otherwise, would inevitably prove suicidal to them.

But, Mr. President, I ask you to look a little further into what individual effort has done for us. I have before named Dr. Solyman Brown. He, sir, was one of the first to break through the trammels which narrow selfishness had thrown around the practice of dentistry. Who is there that can read his treatise on mechanical dentistry, and not feel a generous impulse from the truthful liberality which evidently guided his pen. Another man whose name is more in the ears of the profession than most others, is professor C. A. Harris. Look with me, if you will, at the labor he has performed, both alone, and in conjunction with his colleagues in the college, and on the tripod, in order that the feelings, intercourse and practice of the profession might be liberalized, and made to be known and acknowledged as a liberal profession.

Is there nothing, yea, is there not much, gentlemen, which you all are constantly practicing, which you have derived, directly or indirectly, through their labors. Are there no instruments or modes of practice essential to your success in business, which he or some of his colleagues had the legal right to have patented; undoubtedly there are, but to these, honor is more than money, and respect more than notoriety.

I have before me, sir, the names of many men, whose minds and souls are of a similar mould to these, but time prevents my dwelling longer on this point. I will name but one more member of the profession, and he, only to contrast his previous action with his present, in order, if possible, to convince him that he is inconsistent with himself. I allude to Dr. James Taylor. As you all must now know, he has, this day, stultified himself. He is now upholding and supporting a man who has secrets in his practice and *holds* them for sale.

Now, sir, let me here say, that if there is any one thing Dr. James Taylor has been known for in the west, more than for any other, it is, that he has been willing to communicate freely with all. Especially have I heard this said of him by those who knew him, at least professionally.

Now, sir, let Dr. Taylor pursue the course he has this session commenced, and his honor must inevitably wain. Let the society adopt the course the majority of the committee propose, and I will hold, that it also has, in that act, turned its back on all it has gained. Let it pursue this course, and it inevitably sinks itself in the esteem of the liberal minded.

One thing, all know, who are observant readers; and this is, that the profession has improved its practice very much within the last ten or fifteen years, and that this improvement has, in the main, been brought about, as I have pointed out. But, sir, we are indebted to a class of men, not members of the profession, but who have, nevertheless, manifested a degree of liberality that would put to shame many who claim to be of it. We are indebted to various instrument makers for many of the perfect adaptations of instruments to the several uses to which they are applied. One, I will make particular mention of; Chevalier, of New York, has given us the most perfect file-carrier ever used. He has given us, also, an excellent foot lathe, and a drill stock. He has also made various improvements in forceps. All of which, he could have patented, and prevented others from manufacturing the same. But he partook too largely of the spirit which he knew existed in the profession, therefore he *would not*. He says to his fellow tradesmen, I ask only honorable competition, let the market be with him who makes the best. My improvements I give to the profession, if you manufacture so as to suit them, I am satisfied, I interpose no barrier.

See, also, how much has been done by both manufacturers, to enable us to perfect our artificial work. They have produced much that might have been made the subject of patents, but they would not. They also have said to the profession, buy where you can get the best article, buy where industry has produced the greatest success. And to their co-laborers in the same field, do what we have done if you can, we throw no patent barrier across your experimental path. Yes, Mr. President, they also are trying to teach some of us liberality. Some there may be, who have become too old to learn this lesson. But let me beseech those who have not only learned, but themselves taught this lesson, not now to abandon it and yield the palm in liberality to men who do not claim for their profession, "that it is a liberal profession."

There is still, sir, one other point in my report which I have said nothing upon, and can do little more than remind you of it. This is, that neither your committee or the society have had *any proof* that the change is an improvement. No cases have been presented which have been in actual use in the mouth for *any* length of time.

However beautiful the work may be, (and I acknowledge it looks fine,) I would advise that this society pass no recommendation of it either in the shape of a gold medal, or of the resolution offered by a majority of the committee. I would advise this course, even were there no intention to patent it. One year's practical test will be none too long to try its merits on the mouth. We should, I think, sir, let experience have its due influence on our minds. Let us not be dazzled by appearances. This would not be the first wonderful thing that has vanished, when the practical application has been undertaken. To go no further back, let me ask where is its immediate predecessor, I mean "Dr. Allen's improvement for restoring the contour of the face?" Gone sir, gone into oblivion, because of its impracticability.

Dr. Allen. It is not so, Mr. President, that thing is of just as much value as it ever was, it has not proved impracticable. The very first case in which it was applied, it is still used. It is true I have not pushed that matter much lately, but this was because I have been so much engaged in this matter.

Dr. Leslie. Does Dr. Allen still wear one in his own mouth.

Dr. Allen. Yes sir, I have the improvement now in my own mouth, and if the society desire it I will show it.

Dr. Leslie. I will not, Mr. President, deny what Dr. Allen says, but one thing I must say, Dr. Allen does not look near so fleshy in the face as he did about the time he received that gold medal. "The contour" has evidently changed since that time. This, I think, all Cincinnatians must observe.

But to proceed, let us learn, gentlemen, by the experience obtained from the hasty action of a kindred society, regarding his contour improvement, to avoid the trap before us, and neither reward or commend an *untried* article. And especially one which *we know* will be patented if Dr. Allen can effect it.

When the American Society voted Dr. Allen a medal for his invention of an instrument "for restoring the contour of the face," it done it with the understanding that he presented it to them and the profession at large, on the broad basis of adding something to the common stock of liberality, which the society as a unit were using as a lever, to elevate the profession. And when I say that had that society known or supposed Dr. Allen intended patenting that invention, no medal would ever have been voted him by that honorable body. *I know* I say that which I have the best authority for stating. No, sir, with half the facts we have in this case, they never would have done it in that.

But there are other reasons over and above those given in the report I present, why we should act with caution in this matter.

Even were there no objections to a patent, and that it was fully proven that all that is claimed for the specimens before us is correct, and the practicability of the thing established, still I would be constrained to inquire further into the matter. I would ask you, gentlemen, are *you sure* Dr. Allen is the individual entitled to all the credit given in the preamble and resolution of Dr. Goddard in the majority report. Permit me to say, I think he is not, and I shall as briefly as possible present you my reasons for holding such belief. Premising that I am prompted in all my action in this matter, solely by a desire to do justice to all. I have ever held it to be a very essential principle of professional action, to scrupulously yield to each member of the profession, full credit for whatever I may have received from him, and not allow myself from personal feeling to either under or overrate their actual worth. What I shall now state, I wish the society to know, has already been made known to him by myself, and I now repeat it. In order that if I am in error, the Dr. may have a public opportunity of setting me right, and correcting what is publicly spoken of in Cincinnati. I wish the truth always in all matters, and nothing but the truth.

Dr. Allen, I repeat, is not, in my opinion, (and I have endeavored to form it carefully and candidly,) the inventor of all he claims. Here are my facts on which I base this opinion. I assert first, that it is a fact, that so far as the profession in Cincinnati is concerned, we know nothing of Dr. Allen's experimenting, in order to procure an artificial gum which he could flow around ordinary plate, or other single teeth *until after it had been announced that Dr. Levett had patented a mode of enameling plate work.* *Shortly after* said announcement, Dr. Allen first exhibited to several persons some of such kind of work. This was towards the fall of 1849. These specimens were not satisfactory to the Dr. himself or to those members of the profession who saw them. In these specimens he applied the gum or enamel *after the teeth had been backed and soldered in the usual way.* His only object was to cover what plate was not hidden by the teeth, and to fill up between them so as to prevent the lodgment of food there.

It was owing to these experiments, I suppose, some members of the society suggested at the meeting held at Louisville, September, 1849, that Dr. Allen be appointed to prepare a paper on the subject of artificial gums to be read at our next annual meeting. This appointment the doctor accepted. And how, sir, has he filled it? At the next meeting he was absent; at this one we have no paper, although he has had two years in which to prepare it. But, sir, to pursue the point now before me, I would inform you that about this time, the agent of Dr. Levett visited Cincinnati, he called upon Dr. Allen and and sold him his secret. I have been informed that he purchased the *sole* right to use it in this city and county, and for it paid a good round sum. Liberal, was it not? Whether or not it proved useful to him we know not; but that he continued for a short time endeavoring to flow an enamel on *soldered* work we have reason to believe, but that he met with the success he wished for, at that time, I believe he will not attempt to claim. One thing we in Cincinnati know, is, that in a few months he ceased to mention it. There were too many plates melted, and teeth cracked, for it to be considered an improvement, so the doctor followed again the old mode.

One idea, however, you must perceive he derived from the announcement of Levett's patent, and this the enameling of plate in artificial dentures. This is an essential idea in the mode Dr. Allen claims, and evidently lays at the basis of all his experiments, let the credit be with the inventor of it, viz. Dr. Levett.

Another very important part of the invention I will lay claim to on behalf of another individual, there being not the *least* doubt in my own mind that from him Dr. Allen has directly or indirectly received it.

You are aware, doubtless, that in these specimens presented here by Dr. Allen, that single teeth are set into a base of a silicious nature, which when fused makes them and the base one. Now, sir, I need hardly point out to you, that this thing of using single teeth in this way, is the most important idea of the whole matter. It is the *suggestive* one which prompted the doctor to look around him for an enamel or silicious substance, by which he could produce something resembling that which another person had already effected.

Now, Mr. President, so far as my knowledge of experiment in Cincinnati is concerned, (and I have been careful to keep posted up in such matters as have been in any way made public,) I am *certain* that to Dr. Wm. M. Hunter is due for the credit of first having used single teeth in this way, in Cincinnati. As far back as the spring of 1846, I was aware that Dr. Hunter was engaged experimenting in the improvement of block work, by overcoming the shrinkage and reducing the labor. He then showed me specimens of what he had accomplished in this way. And one fact he *then* explained to me was, *that he could insert single teeth in a base*, (silicious of course,) and fix them there by fusion of the base, thus doing away with much carving and overcoming part of the shrinkage. I have been shown several of his specimens occasionally from that time to this. Dr. Hunter has several modifications of the same principle. One of them is to mould his base, as he would in ordinary block work on his gold plate, the teeth are then arranged in this base, this is removed from the plate, placed in a muffle and subjected to a fusing heat. They are then ready to be attached to the plate which is done by backing and soldering as ordinarily followed, or with the continuous backing.

Another mode is to take a piece of thin platina plate, say six or nine lines broad, and the length of the ridge of the mouth, this is notched on the edge so that it may be bent into the curve of that part of the alveolar ridge on which the base of the teeth sit, it is then struck up over the gold plate, the inner edge is notched so that it may be bent up behind the teeth, these parts are punched so that the lower pin may be inserted; this, when riveted, holds the teeth in place. A base or body is now filled in around them, and the whole subjected to a fusing heat; when removed from the muffle, the platina and base are united; this is next placed on the gold plate, gold backings are now attached to the upper pins, the surfaces of the gold and platina plates are boraxed where they come in contact, solder is applied, and the whole placed in a muffle and the solder fused, resulting, Dr. Hunter informs me, in a perfect union of the gold and platina plates. Specimens of both these styles, the doctor assures me, he has sent to the world's fair. That Dr. Hunter sent specimens there, the profession in Cincinnati, and I suppose elsewhere, know full well, for it was announced in the published lists months before Dr. Allen exhibited any specimens of his recent improvement. The specimens of Dr. Hunter were also a subject of professional conversation in Cincinnati, he having shown them to several persons before sending them to London, and was not until months after they were sent away that Dr. Allen produced any thing like them. This then, gentlemen, I believe to be another important reason why Dr. Allen should not have a gold medal, or have this thing recommended as his improvement.

But, gentlemen, this is not all. Circumstances which have have more recently transpired compel me still further to question his claims, a portion of what I shall now state is *fact.* From this I have drawn some inferences; the first, I trust, you will believe, the inferences you must judge the correctness of. The individual whom I shall presently name, makes assertions, these you must estimate for yourselves. My object in alluding to them thus publicly is to do that which I have done privately to Dr. Allen, give him an opportunity to prove that false which is publicly stated in Cincinnati. The profession in Cincinnati have been waited upon by a gentleman by the name of Steemer, with an improvement similar to the one before us, offering to teach the profession for a consideration, the method of manufacturing the enamel and gum, and of setting teeth just in a similar manner to that of the specimens of Dr Allen.

This I know to be a fact, for he showed me specimens of his work. I will not say it was done in such a workmanlike manner as a dentist might do it, but the principle evidently was there. True it was, that the arrangement of the teeth was bad, but that was nothing when estimating his knowledge. What he had for sale was his knowledge of enamel, and the *mode* of operating.

As to the capability of that he showed me being able to resist acids, I am satisfied. And the specimen I tested being on silver, (he also showed me some set on platina,) it must of course have been largely composed of glass of borax. Hence, that such as could be used on platina or gold, would be able to stand the fluids of the mouth, I feared not. It was that I had put a portion of his specimen into concentrated sulphuric acid, without producing change in it, that made me indifferent to experiments proposed in committee, with acid tests of those specimens of Dr. Allen.

This, Mr. Steemer tells us in Cincinnati, without the least hesitation, that he has sold this receipt to Dr. John Allen, and also to Dr. Darling. From the latter, he holds a certificate of that fact. And that he rendered some services to Dr. Allen, he, Dr. A., has admitted to myself. Dr. Allen, it is true, denies that Steemer has taught him this mode of setting teeth, or that said Steemer invented it. This, sir, is denying more than I have any idea of claiming for him, for you will perceive I have already set aside the leading and chief points to two other individuals. I know not, sir, that for Mr. Steemer, I will positively claim any thing, until I have further proof of his assertions. But, sir, from some *facts* I will draw some inferences. If these inferences are incorrect, Dr. Allen will oblige me much, and serve his own cause, by proving them so, and that the *facts* are misunderstood. I know I consult his own good, however much I may risk my own immediate welfare, by giving him this public opportunity of proving that to be false which some suppose to be true. Truth is what I suppose we all want on all subjects, and it is my love of it and justice, that prompts me to stand here alone, I fear, on the side I am. But to the facts and inferences. This Mr. Steemer spoken of, is a German by birth, and has been reared an enameler, and public reputation accords to the Germans a creditable standing in that occupation. He was induced some few months ago, to enter into some business arrangement with Dr. Allen; what the precise nature of it was, I am not fully able to say. I would not presume that he engaged to teach the doctor dentistry. But, Mr. President, we in Cincinnati, know that it is only two or three months since Dr. Allen first showed specimens of *this kind* of work, possessing whatever degree of perfection it may now have. And that this improvement has been all made, so far as the profession in Cincinnati know, *since* this gentleman formed a connection with the doctor. It is also a fact, as has been stated by Dr. James Taylor, in the last number of Vol. 4, Dental Register, in his remarks on Dr. Allen's *first announcement* of his improvement. "That we regard the color of the gum which the doctor uses, as much improved since we first noticed it."

This improvement of the color, be it remarked, was also made after Mr. Steemer and Allen's connexion.

From these facts, sir, others as well as myself, have inferred that Mr. Steemer must have been of essential service to the doctor. Not that Steemer had ever set teeth in this way before he saw Dr. A., or that he suggested the same to him, I have, I think, shown you that suggestion the doctor received from another source. What then was that service. The doctor, I infer, possesses as the result of his former experiments the knowledge of an enamel, in the use of which he could not obtain a good gum color, probably by combining in it a good share of glass, containing metallic oxyde, (lead or zinc,) which changed the gum. Now, Mr. Steemer having been accustomed to prepare enamel for culinary vessels, in which was required

*great cohesion to a metallic surface*, combined with the power of *resisting acids*, and the *absence of oxydes of the baser metals.* I infer, that of him the doctor obtained a knowledge of the *materials* he had been "*so long*" in quest of. In a word, that Mr. Steemer gave to Dr. Allen the knowledge which *enabled* him to make a profitable use of the two other principles which he received of Drs. Hunter and Levett. This, sir, I contend, is a just inference. It is the most natural one to be drawn from the facts of the case.

If I have been misinformed as to the circumstances, it should be easy for Dr. Allen to prove his position. Until he does so, I must view Dr. Allen as claiming that which he has no title to, and for one, reject the proposition of the original mover, and the majority report.

But, Mr. President, I am not disposed to leave the matter without a word further. I have said much, I know, which detracts from the claim of Dr. Allen, but I am conscious I have endeavored to do it justly.

There is one feature of these specimens which I confess *is new* to me. This is his *depending upon the adhesion of enamel to the plate, as sufficient to retain the teeth as firmly as when soldered on.* If this, after sufficient *practical test*, is substantiated, I will say that *this* is a valuable idea added to our stock of knowledge. And to Dr. John Allen will I give credit for *it.* But, sir, to this, he must grant time. Let us be cautious, remembering that more harm results from precipitancy than the opposite.

Dr. James Taylor. Mr. President, I am willing to confess that my mind has been changing for sometime on the subject of patents. It is true, I have heretofore stood, as the extracts read by Dr. Leslie show, and I need not refer to them again, to show where I have formerly stood, Dr. Leslie has done this so well, that I am saved that trouble. My views on the subject of patents, now is, sir, that it would be best to grant this right to the members of the profession, so far as regards the patenting of any thing strictly mechanical. But, sir, I would be as much opposed as I ever was to the patenting of any thing not mechanical, to the patenting of any thing in our profession, calculated to alleviate the sufferings of poor humanity, in the treatment of disease. The man who would do this, would be unworthy of professional fellowship.

Dr. Leslie. I would ask the doctor to explain, when he says he would not agree to the patenting of any thing not mechanical, does he mean that any medicinal compound preparation, or simple, calculated to remove or relieve disease or suffering in the mouth, should never be patented, but that all of dentistry aside from this, may be.

Dr. Taylor. Yes, sir, that is what I mean. This course may be pursued without injury to our patients, and leaves us room to encourage those who are indefatigable in their labors to improve the practice of dentistry. This principle is allowed by the members of the medical profession. It is but a few days since I was shown a stethoscope, invented and patented by a practicing physician of Cincinnati.

It is viewed as a valuable improvement, so much so, that I think no physician will, hereafter, make an examination of the lungs without this improved instrument.

Dr. Leslie asked, if the inventor is a member in good standing in the medical society.

Dr. Taylor. I believe he is, and he is known to be the inventor. Besides, this is no new thing for physicians to do. There are trusses innumerable, and very many supporters which have been patented by respectable members of the medical profession, and which are recommended by the eminent physicians.

There has also been numberless surgical instruments secured to the inventor by patent. So that you see, sir, the position I now stand in ; with reference to patenting, is that allowed and upheld by the medical profession.

That to Dr. Allen should be granted the right to patent, seems to me but justice, having, as he says, devoted a great deal of time and money to the development of this important improvement, for important I believe it to be. It would be expecting too much that a man should devote years of study and labor without a reward, and I think, Mr. President, it is the least we can do in this case, to adopt the proposition of the majority of the committee, and recommend to the *profession* this improvement.

Dr. Leslie. Mr. President, it makes me smile to hear Dr. Taylor speak as he does, so very ridiculous does his position seem. It reminds me, sir, of the old story of the Irishman's gun. *Now*, the Dr. is willing that if you protect poor suffering humanity from the evil effects that might flow to them from granting to members of our profession the right to patent the medicines they may make use of in dental practice. Why then he rests satisfied. Cut off the right to the patent use of that the Dr. has little use for, very little use for, I may say. As the treatment of disease in the mouth by him or dentists generally, beyond what may be successfully treated by an astringent or stimulant, or the lancet, seldom occurs. Grant this, I say, and he is willing you may patent anything applying to the whole or a part of the balance.

And yet, the Dr. would have us believe he is still contending for something grand, something worthy of a liberal member of a liberal profession. It is this makes me smile. It is this reminds me of the Irishman and his gun in need of repair. He said it wanted a new stock, yes, and a lock, aye and a barrel also ; when fully examined, his gun only consisted of the flint. Now, sir, I hold, the Dr. is in a worse plight than the Irishman. Let him deduct from his practice, that portion in which he uses a medicinal preparation and he will find the value of such portion exceedingly small.

He will, I feel confident, have not enough to form half a flint. Sir, it is idle for him to attempt to lead us astray in this way. He now follows only the *shadow* of a resistance to patents. He has in fact yielded the whole ground he once occupied, having some reason, therefore, which you must have noticed he has not even attempted to state, although I have called upon him for his reasons, and do now again urge upon him again, for I have no desire to stand alone in this society. If he has sound reasons and correct motives to influence a change in my action, I pray him to make them known. Until these are produced, I must hold the doctor has made a very lame defence.

Dr. Allen. I know not, Mr. President, that I am entitled to speak in this matter just now, as so much time has been consumed already.

Dr. Leslie here remarked, that he hoped Dr. A. would be heard with all patience, and be given unlimited time, for any remarks he may have to present, inasmuch as his claims had been explicitly denied him.

Dr. Allen resumed, and said, that it always had been and supposed would continue so, that so soon as an individual presented something of value to the public, there would be found those who stood ready to rob him of all the honors that attached to him. Of this man Steemer, he would say, there was no truth in his statement that he had taught him. Just look at it, here comes an ignorant Dutchman, a man that knows nothing of dentistry, and claims to have taught me how to set teeth in this highly elegant manner. Sir, there is no truth whatever in it, I have got no valuable idea from him. The true history of his connection with me is simply this, I had been engaged for years at a cost of much labor and time experimenting in this thing. Owing to a press of business in which my own and my students' time was fully taken up, I could not find time to pursue my investigations in this matter as steadily as I wished; I, therefore, at the suggestion of one of my students, who was acquainted with Steemer, and knew in what he had been engaged, employed him, and put him in my laboratory, that he might assist me in this matter by grinding materials, &c. to which I could not spare the time of either of my students. When there, of course he had an opportunity of ascertaining what I was after. We little supposed at the time, he was stealing from me the principle on which I do this work, but so it has since proved. He was there as a mere assistant, and for his services rendered, I paid him *well*, and it is hard if he is to step in and rob me of my dearly earned rights. Why, he never set a tooth in his life, and never saw teeth set in this way until he saw it in my laboratory. He never attempted it until after he left me, and now he is going around offering for sale what he has stolen from me. And some people will rather pay him for his stolen goods than come to me. The reason is, they are not willing to allow me the credit.

But, Mr. President, the thing, this man Steemer says, is the same as mine, is it not? although he stole a good deal more than I had thought at first. He, however, cannot do or teach any one this kind of work. He has as yet sold it to only one individual, (Dr. Darling, of Cincinnati,) and he is satisfied that it is perfectly useless, and has applied to me to be taught my mode, and he is now waiting my return, in order that he may obtain a knowledge of it. Why, sir, what has this man been that he should set himself up to teach dentistry? only an enameler of pots, nothing more. I have not, sir, come prepared to meet the matter as it has come before us, if I had thought it necessary, or were there time now, I could produce affidavits from my students, to show that I have done this thing before I ever saw Steemer, but I did not think that any one was going to lay claim to it. As to Levett's enamel we all know, that it is of no use whatsoever, so that he has not helped me any. As to Dr. Hunter's improvement, I contend there is no similarity between them. This is entirely different from what he has done, so I have nothing more to say on that head. But it has been said I have no right to patent this thing. Why, sir, this would be a pretty way to encourage men to spend their time and money to improve and elevate the profession; just so soon as he has obtained any thing of worth, he must give it all for nothing.

He may have pursued it after others had been hours in bed, still, he must plod away, and spend vast sums to discover this thing, and then not get any return. It is, sir, I think, essential to the improvement of the profession, that we recognize the right to patent improvements. This course has been found necessary by our wisest legislators. They have enacted laws for the encouragement of national effort, and the protection of individual right to inventions. Our own government has established a separate department to watch over this matter, and unless we agree to do the same, the result will be, that every man will keep his discoveries to himself.

Dr. Goddard had little to say on the matter.

He thought it evident, that we must encourage all meritorious discoveries, and we could not expect that an individual having made great sacrifices of time and money to make a great improvement, should give this to the public indiscriminately, without any reward. And until such time as we obtain some such system of national rewards as exists in France, we must grant the right to patent such improvements as this. We know that Dr. Allen has been engaged for a long time, and spent much money experimenting in the matter, and now, that he has arrived at perfection, I think it is as little as we can do, to pass this resolution, offered by the majority of the committee.

Dr. Leslie. Mr. President, it may be right for Dr. Allen to use those words, stealing and stolen so frequently as he has, but it does sound to me very harsh on this floor, (Dr. Allen remarked, best to call things by their right names,) the Dr. may be justified in it by the facts, but I doubt it. That the Dr. has not denied any of the *facts* I stated, I suppose, must have been observed. That he denies only one of my inferences is another point, I wish you should notice. He brings no *proof* to his support; he deals only in assertion, and speaks of what he can produce, if time is afforded. This proof, I will anxiously wait for, hoping, for the doctor's sake, he will present it soon.

There is one other point, I wish to make on his remarks, as to the necessity of granting him a patent, that he may be enabled to reap a return for his labor and money. He says, that unless he obtains a patent, he will be robbed of both money and fame. In this, he allows his fears to carry away his judgment. *That* is, *if* some of his statements be correct. In the first place, he tells us, that a necessary knowledge of his mode can only be acquired, successfully, through the *personal* instruction of some one who understands it. Now, as he says, that Mr. Steemer does not know his mode, and as we find the Dr. so careful not to communicate any of the secret to us, I infer, that no one knows it but the Dr. and such others as he may have communicated it unto.

Of course, then, the secret laying in himself, to secure himself, he has only to sell it, with the understanding that they teach no one for or without a consideration. Let him pursue this course, and you perceive, he has the control of the market in his own hands, just as effectually without, as with a patent.

But, sir, I will remark further, before I take my seat, that the gentlemen who have opposed the views I have presented, have given us no reason for retiring from their first position, by awarding the gold medal. The *same* gentlemen, be it remarked, who moved and seconded that measure, now bring a resolution, cutting down, very materially, the honor they first intended conferring on Dr. A. They have reduced it to a simple commendation, and this course they adopt, be it noticed, *before a single word of* discussion has taken place, and now, they give *no* reason for it. Verily, they abandon their friend very unceremoniously. Truly, he may have cause to say, "save me from my friends."

I cannot imagine the cause of this change of tactics. I *know* it could not be the *cost* of the medal, for this the Dr. tells me "he will spare the society, (it being poor,) should it see fit to vote it him." Gentlemen, if you believe, as your preamble and resolutions say, why, sirs, vote him the medal at once, it will cost you nothing. Mr. President, I have done with this matter for the present. I am willing the minority report now go to vote. I may stand alone, but, truth compels me to vote for it if no other member does.

The President now took the vote on the acceptance of the minority report. A majority of the members voted in favor of its acceptance, thus entitling it to appear on the published minutes.*

The motion which had previously been offered, to adopt the majority report, was now renewed, and the vote taken, resulting in eight yeas and one nay. Dr. Leslie here requested that the vote be recorded, with the names attached. This was re-

*I perceive that Dr. John Allen, the Secretary, or Dr. James Taylor, the Editor, has, without any right or justice, stricken this report from the published minutes of the Register. For this, I shall, at the proper time, call them to an account.

fused by the chair, who, together with Drs. Goddard and Taylor, held, that it would be equivalent to taking the vote again. The only way to reach it now, was by a vote to reconsider the matter, or a motion to record the names.

Dr. Leslie held that this was a mere evasion. All *knew* what the vote had been, no one voted against the motion but himself, all he asked, was that the names be recorded; he hoped gentlemen had not done that they were ashamed the profession should become acquainted with. If they had done so, they had better retrace their steps at once. Inasmuch as he had not voted in the affirmative, it was not in his power to move a reconsideration, and as no one who had, offered to do so, the other course only was left open. He, therefore, would move that this vote be recorded.

Dr. Goddard said he was willing that his name might be attached to his vote, he would, therefore, second the motion of Dr. Leslie. The society now voted on this resolution, but it was negatived.

Dr. Goddard arose, and said he had a motion to offer. He remarked, Dr. Leslie thinks he has got us just where he wants us. Ah, he says to himself, I have you, now I will keep you to it. He thinks we have no way to get away from him, but in this he is mistaken, as he will perceive. I now, Mr. President, offer the following:

*Resolved*, That the preamble and resolution which was referred to the committee, with all debate attending it, be expunged from the minutes, and the report offered by the majority of the committee, be substituted in its place.

This resolution was promptly seconded, and Dr. Taylor remarked, that it had appeared evident from the beginning that Dr. Leslie had determined to force this matter upon them. Now, the fact was, that it had been introduced in the form it was first presented in, without due consideration, and although I seconded the original motion, it was not, therefore, evident I was in favor of it. Upon reflection it appeared to me best to change the method of recommending this improvement, and in order that the minutes should present a proper appearance, I

think it best to expunge the original preamble and resolution, inasmuch as we have adopted another course and so that there will not appear anything conflicting in the published minutes.

Dr. Leslie. Mr. President, I understand this move perfectly, it is simply an attempt to cover up that which, I am satisfied, some are ashamed of. It is an idle attempt to place their light under a bushel. But let them be warned not to attempt that which they cannot effect. Their *acts* are now *facts*, and all they can do cannot make them less. Let me say to them, that if they expect by this resolution to expunge their course from my memory, such an attempt is utter folly. Or if they expect by it to tie my tongue and prevent my speaking of their acts, that is also folly. They will find it better, far better to allow the minutes to stand as they are. If they have done that which, on consideration, they have discovered to be wrong, why not acknowledge it like men. It would be nothing new. Wise men it is said, change often, fools never. Besides, some of their acts will become known, but their not appearing on the minutes, some will be left to surmise respecting them, and as surmise is generally worse than reality, they will, if they pursue their course find that their last state is worse than the first. Further, sir, the charge that Dr. Taylor throws out against me of having from the first forced this course upon them, I deny utterly, there is not, sir, any truth in it whatever. That you may see this, let me review the history of yesterday's session, and you will see where he should have laid this charge.

First, we had the short address of Dr. Allen on his improvement. Immediately after this, Dr. Goddard rose and read the paper he had prepared, viz. the original preamble and resolution. To this, Dr. Taylor pretty promptly responded with a second. Then, without a minute for discussion, or a word said, the president was proceeding to take the vote. At this juncture, I arose and arrested that act, desiring to discuss the matter before the vote should be taken, this, of course, could not be denied me. But being too near the hour of adjournment to enter into the discussion to the length I expected, I at once moved the resolution be laid on the table until the afternoon session. In the afternoon we met: the chairman declares the first business to be the consideration of this resolution. Dr. Taylor rises and says he would like to withdraw the original resolution, and offer one he held in his hand. To this, I objected, on the ground of its not being according to correct order. I held that a resolution could not be withdrawn unless mover and seconder united in the request, and the body consented. The chair thought differently, and decided the seconder could withdraw his second, and that if he did so, the resolution was no longer before us. Dr. Taylor said he would then withdraw his second. To this, I also objected, and held, that after a resolution was moved and seconded, and announced from the chair, it ceased to be the property of the mover and seconder. That they had no control over it, that they could not, either singly or combined, withdraw the motion, without the consent of the body, or that either mover or seconder could withdraw said motion or second. This position not only the chair ruled to be wrong, but he was supported by Dr. Taylor and several members, and so evident was it that the majority differed with me, that had the decision of the chair been appealed from, I have not the least doubt the chair would have been supported.

It then occurred to me that, inasmuch as Dr. Goddard, the mover, was not present, it was a courtesy due him by the society, that his motion should not so unceremoniously be set aside without his presence. This I named to you, and its justice at once perceived, and the further consideration waived until he might be present. When he came in, Drs. Taylor and Griffith's position was stated by the chair, when Dr. Goddard arose and said, "that Dr. Taylor *could not* withdraw his second. He might, if he chose, offer a substitute, or as many amendments as he pleased, but the original resolution he could not withdraw, without the consent of the society. That a motion, as soon as moved and seconded and announced from the chair, it became the property of the body, and could not, by the combined or separate action of the mover and seconder, be withdrawn without the leave of the body. This, he was *positive*

was according to Jefferson, and governed in all well managed societies." Now, sir, this was exactly what I held a few minutes before, but was overruled and opposed in. But, no sooner does Dr. Goddard state it so positively, than the same men who opposed me, yield all up to him, and that *without any discussion*, and *without one item of proof*. Now, sir, I think, if any one *forced* this matter on Dr. Taylor and the society, it was Dr. Goddard.

But, Mr. President, if Dr. Taylor should suppose, that since the gold medal resolution has been brought before us, I am desirous of keeping it there, let me assure him, that, in that he would be correct. I have had no hand, whatever, sir, *in bringing this matter here*, but, since he and his colleagues have put it upon the minutes, I am determined to do all in my power, that is proper, to keep it there. I want the profession to know what they have been willing to do; and the Dr. should thank me, that I prevented the vote from being taken so promptly as it was about to be. By my course, he was afforded time for reflection, and saved from a serious mistake. No, gentlemen, seek not to bury the record of your acts. What you have done, if it is right, stand up to it. If it is wrong, acknowledge it, and if you sincerely repent, it may be forgiven. One thing that prompted me to be somewhat tenacious in holding on to correct order, was the improper and unfair means Dr. Taylor made use of to get rid of the resolution. Look at his course in committee. This morning when I reached the hall, no report was shown me until I had asked for it. When obtained, I soon perceived that he had made a change he had no right to do. I found he had copied off the lengthy preamble which had been referred to the committee, certainly an unnecessary labor, the original being plainly written. But his object appeared at the foot of the page. I found that he had, on his own responsibility, expunged Dr. Goddard's resolutions awarding the medal, and to make it look the original, he had inserted the following: "Therefore, *Resolved*, That we appoint a committee of three, to examine said specimens." This, sir, I at once objected to, and insisted on the correction. In this, Dr. Goddard concurred, and he inserted his original motion and scored out the other. This, gentlemen, you can call by what name you think best. That I detected and corrected action on him, which I thought him above, is most certain. It was then evident to me, why he had not offered to show me the report, or ask my signature to it. You can judge now, who has sought to force matters.

I defy him to show, that I have for one moment, deviated from that which was strictly correct and according to truth. All I have done, I am willing all men may know and judge, will others do the same? Before I sit down, I will, to test this again, move, that when we vote on the expunging resolution, we shall do so by calling the roll, and that the vote be entered on the minutes.

This, Dr. Goddard seconded, and the vote being taken on this motion, it was adopted. The question being now called for on the expunging resolution, Dr. Goddard said, that, inasmuch, as the vote on this resolution would, undoubtedly, be the same as the vote on the adoption of the majority report, he would propose, if it would better satisfy Dr. Leslie, that we would consider the vote taken on this resolution as that taken on the other, and that the names obtained as the vote on this, should be inserted as the vote on the majority report, and attached thereto.

Dr. Leslie remarked, this would suit him very well. It was what he had tried to get gentlemen to do before. The roll was now called, and the following was the result :

*Yeas*—Drs. Griffith, Goddard, Taylor, Baxter, Ulrey, McCallum, Davis, A. M. Hunt.

*Nay*—Dr. Leslie.

This, Mr. Editor, was the end of the discussions.

The meeting, after attending to some small matters, adjourned to meet in Cincinnati next year.

I have sent you this report in order that it may have a wide circulation, and that the profession may know, through the pages of the Journal, what we are doing and saying out here in the west. A. M. L.

